# Physiological and Biochemical Responses, and Comparative Transcriptome Profiling of Two *Angelica sinensis* Cultivars Under Enhanced Ultraviolet-B Radiation

**DOI:** 10.3389/fpls.2021.805407

**Published:** 2021-12-17

**Authors:** Tong Peng, Yinquan Wang, Tao Yang, Fusheng Wang, Jun Luo, Yali Zhang

**Affiliations:** ^1^College of Pharmacy, Gansu University of Chinese Medicine, Lanzhou, China; ^2^Northwest Chinese and Tibetan Medicine Collaborative Innovation Center, Lanzhou, China; ^3^Key Laboratory of Microbial Resources Exploitation and Application, Institute of Biology, Gansu Academy of Sciences, Lanzhou, China; ^4^Dingxi Academy of Agricultural Sciences, Dingxi, China

**Keywords:** *Angelica sinensis*, UV-B radiation, transcriptome, physiological response, antioxidant defense system, bioactive compound

## Abstract

In this study, we explored the adaptive mechanism of two varieties of *Angelica sinensis* exposed to enhanced Ultraviolet-B (UV-B) radiation. The radiation had different effects on the biomass, photosynthetic performance, oxidative damage, antioxidant defense system, and levels of bioactive compounds of Mingui 1 (C1) and Mingui 2 (C2). C2 outperformed C1 under enhanced UV-B radiation, compared to natural light. Using the Illumina RNA-seq, we obtained 6,326 and 2,583 DEGs in C1 and C2, respectively. Under enhanced UV-B radiation, the mRNA levels of genes involved in photosynthesis, antennae protein synthesis, carbon fixation, chlorophyll synthesis, and carotenoid synthesis were decreased in C1 but stable in C2, involving few DEGs. TFs were widely involved in the response of C1 to enhanced UV-B radiation; almost all *bHLH* and *MYB* coding genes were downregulated whereas almost all genes encoded *WRKY22*, *WRKY50*, *WRKY72*, *NCF*, and *HSF* were upregulated. These results indicate that enhanced UV-B radiation was not conducive to the synthesis of flavonoids, while disease resistance was enhanced. Regarding the ROS scavenging system, upregulated DEGs were mainly found in the AsA-GSH cycle and PrxR/Trx pathways. Remarkably, DEGs that those encoding biosynthetic key enzymes, including ferulic acid (*CHS*, *CHI*, *DFR*, and *ANS*) and flavonoid (*CHS*, *CHI*, *DFR*, and *ANS*), most upregulation in C2, leading to increased accumulation of ferulic acid and flavonoids and adversely affecting C1. Genes encoding key enzymes involved in the synthesis of lactone components (*ACX*, *PXG*) were mostly up-regulated in C1, increasing the content of lactone components. Our results reveal the DEGs present between C1 and C2 under enhanced UV-B radiation and are consistent with the observed differences in physiological and biochemical indexes. C1 was more sensitive to enhanced UV-B radiation, and C2 was more tolerant to it under moderate enhanced UV-B radiation stress. In addition, the large amount of *A. sinensis* transcriptome data generated here will serve as a source for finding effective ways to mitigate UV-B enhancement, and also contribute to the well-established lack of genetic information for non-model plant species.

## Introduction

Radix Angelica Sinensis (RAS) is the dried root of *Angelica sinensis* (Oliv.) Diels, which has been used as both a medicine and for nourishment in the form of spice and tonic for more than 1,000 years in China, South Korea, and Japan. Today, it is still one of the most commonly used herbs by practitioners of Traditional Chinese Medicine (TCM) in China as well as Europe (Wei et al., 2016). More than 70 active compounds have been identified in RAS, including polysaccharides, ferulic acid, coniferyl ferulate, Z-ligustilide, E-3-butylidenephthalide, and other phthalides (Hook, 2014). These active compounds have numerous pharmacological actions, including anti-inflammatory, antitumor, immunostimulatory, hormone regulation, antihepatotoxic, neuroprotective, and anti-aging effects, among others (Chao and Lin, 2011).

RAS is mainly cultivated in the alpine wetland of southeast Gansu Province of China, in the northeastern margin of the Tibet Plateau. *A. sinensis* prefers a cold summer climate, sufficient rainfall, low light, and loose, organic-rich loam soil. In the past 10 years, due to global climate warming, and severe early bolting and root diseases, the planting area of *A. sinensis* has gradually changed from low-altitude mountain valleys to high-altitude slopes; thus, it is now being exposed to enhanced UV-B stress (An et al., 2020).

**FIGURE 1:**
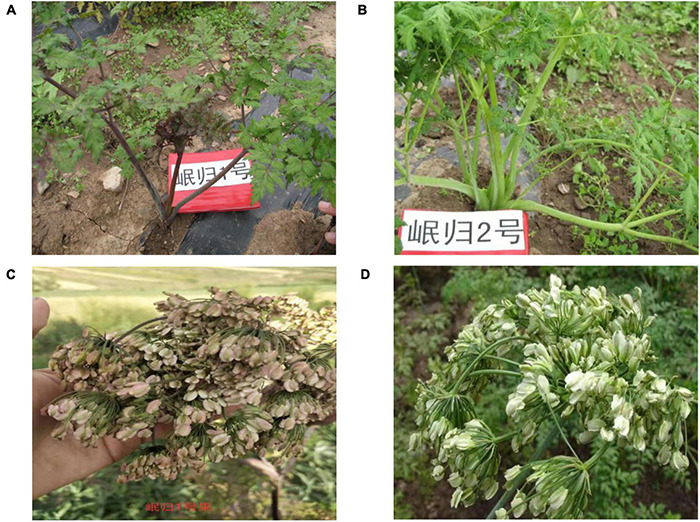
Morphological performance of **(A)** Mingui 1 (C1) plants, **(B)** Mingui 2 (C2) plants, **(C)** C1 flowers and fruits, and **(D)** C2 flowers and fruits.

Mingui 1 (C1) is a popularized *A. sinensis* cultivar with dark purple petioles, dark green leaves in the growing period, and light purple flowers in the flowering and fruiting period. It has been cultivated throughout the planting area of *A. sinensis* but has suffered from problems such as biological contamination caused by agricultural residues and pathogenic microorganisms, and cultivar degradation, and so forth. Mingui 2 (C2) was bred from the mixed population of cultivated *A. sinensis* with combining methods of group breeding and systematic breeding by Dingxi Academy of Agricultural Sciences, Gansu Province, China. C2 shows a typical characteristic in its stem and leaf is light green leaves and petioles during the growing period; green stalks with white flowers, and light yellow and white seeds in the flowering and fruiting period. Nei’s genetic of between Mingui 1 and Mingui 2 was 0.0690, and both were clustered by UPGMA into the same class at the genetic distance of 0.10, which indicated there were great genetic differences among different *A. sinensis* cultivars (Yan et al., 2014; Figure 1).

Ultraviolet-B (UV-B, 280∼315 nm) is a natural component of sunlight, and a small amount of it can have a great impact on organisms (Robson et al., 2019). Generally, there is a positive correlation between the UV erythemal dose and altitude (Xiao et al., 2019). The impact of UV-B on plant growth and development is largely dependent on radiation dose and plant species (Jenkins, 2009). Both UV-B-specific and non-specific pathways may be activated, leading to damage of photosystem II (PS II) and photosystem I (PS I), the loss of thylakoid membrane integrity, a decrease in chlorophyll level, and carbon fixation (Nawkar et al., 2013). The accumulation of reactive oxygen species (ROS) may damage cells, proteins, lipids, and DNA, and further damage the photosynthetic apparatus (Jansen et al., 1998). It can also have adverse effects on stress-related hormone synthesis (Mosadegh et al., 2019). A low dose of UV-B radiation can induce a photogenic morphological response and UV-B adaptive response in plants (Yin and Ulm, 2017), leading to an increase in chlorophyll levels (Vandenbussche et al., 2018) as well as antioxidants such as superoxide dismutase (SOD); peroxidase (POD), catalase (CAT), and nicotinamide adenine dinucleotide phosphate (NADP) dehydrogenase, and non-enzymatic defense systems such as glutathione, polyphenols, tocopherol, and ascorbic acid (Yu and Liu, 2013). At the same time, UV-B-absorbing metabolites, including flavonoids and other phenolic substances, will accumulate (Inostroza-Blancheteau et al., 2016). Some transcriptome studies have shown that UV-B regulates different functional genes in Asian ginseng (Panax ginseng) (Gong et al., 2020), Lycium ruthenicum (Chen et al., 2015), and other medicinal plants. This includes genes related to the photosynthesis pathway and photosynthetic pigment synthases, such as *Lhca*, the *Lhcb* gene family, and the Psb complex (Kataria et al., 2014). Transcription factors (TFs) are also involved in the plant response to UV-B stress. Among them, WRKY, MYB, NCF, and *bHLH* are the most well-known and are considered important regulators of UV-B response genes in plants. Hormone-related genes, such as those encoding biosynthesis and signal transduction pathways of indoleacetic acid (IAA), gibberellin (GA), cytokinin (CTK), jasmonic acid (JA), and salicylic acid (SA), are involved in the adaptive response of plants to UV-B stress (Vanhaelewyn et al., 2016). These include many structural and regulatory genes in the phenylalanine/flavonoid pathway, such as phenylalanine ammonia-lyase (*PAL*), chalcone synthase (*CHS*), dihydroflavonol 4-reductase (*DFR*), and flavanone-3-hydroxylase (*F3H*) (Neugart et al., 2014; Henry-Kirk et al., 2018). Other genes encoding lactone component synthase are less well studied.

As discussed above, the understanding of UV-B effects has shifted from general stress factors to specific regulators to promote metabolic and developmental changes in higher plants (Parihar et al., 2015). Previous studies on the growth and development of *A. sinensis* have mainly focused on the mechanism of early flowering (Yu et al., 2019; Gao et al., 2021), or the effects of rhizosphere microorganisms on the quality of *A. sinensis* under different soil microenvironments (Zhu et al., 2021). Some of these isolated and identified autotoxic allelochemicals from the rhizosphere soil (Xin et al., 2019). The effects of enhanced UV-B on different *A. sinensis* cultivars and the responses of physiology, metabolites, and comparative transcriptome have not been sufficiently studied.

Therefore, in this study, we investigated the physiological and biochemical responses of two *A. sinensis* cultivars to exposure to enhanced UV-B radiation as well as the underlying molecular mechanisms.

## Materials and Methods

### Plant Material and Enhanced Ultraviolet-B Radiation Treatment

Two *A. sinensis* cultivars [Mingui 1 (C1) and Mingui 2 (C2)] were used. Seedlings were obtained from the Dingxi Academy of Agricultural Sciences in Gansu, China. The experiment was run in Min County, Gansu Province (34°59′ N, 103°57′E, 2,340 m above sea level) from April 2019 to November 2019.

Enhanced UV-B radiation treatment was carried out using an adjustable UV-B lamp (wavelength range: 280∼400 nm) set 0.25∼0.5 m above the top of the plants (Supplementary Figure 1). The stress intensity of UV was set at two levels, natural light (U0) and the radiation dose equivalent at 2,600 m above sea level (or 14.11 kJ⋅m^–2^⋅D^–1^, Ut). Hence, the four treatment groups were: C1 plants exposed to U0 (U0C1), C2 plants exposed to U0 (U0C2), C1 plants exposed to Ut (UtC1), and C2 plants exposed to Ut (UtC2). Each treatment was repeated three times. Treatments started on July 5; for each treatment, the UV lamp was turned on at 8:30 a.m. and turned off at 5:30 p.m. After *A. sinensis* leaves were collected, they were rinsed with distilled water, immediately frozen in liquid nitrogen, and stored at –80°C for further analyses including physiological and biochemical index determination and high-throughput sequencing.

### Photosynthesis Parameters

The gas exchange parameters, including photosynthetic rate (Pn), transpiration rate (Tr), and stomatal conductance (Gs), were recorded using an infrared gas analyzer (IRGA) (Li-Cor) between 09:00 and 11:00 a.m. We used an LI-6400–40 portable photosynthesis system (Li-Cor, Lincoln, NE). The CO_2_ concentration was set at 350 μmol⋅mol^–1^, the light intensity was 1,000 μmol⋅m^–2^⋅s^–1^, and the temperature was 28°C. Each leaf was measured three times and the average value was calculated. Water use efficiency (WUE) was calculated as the ratio of Pn/Tr.

### Photosynthetic and Photoprotective Pigments

Samples (0.25 g) were ground in liquid nitrogen and incubated in 2.5 mL 80% acetone at 4°C in darkness. The levels of chlorophyll (Chl a), Chl b, carotenoids (Car), and total chlorophyll (TChl) were determined using a UV-vis spectrophotometer (Cary-50), measuring the optical density (OD) of leaf extracts as previously described. Car was measured using a Car measurement kit (Qincheng Bio, Shanghai, China), according to the manual.

### Antioxidant Activity

According to the manufacturer’s instructions, the levels of Superoxide dismutase (SOD), peroxidase (POD), catalase (CAT), and malondialdehyde (MDA) were measured with commercially available SOD assay kits, POD assay kit, CAT assay kit, and MDA assay kit (Jiancheng Bioengineering Institute, Nanjing, China), respectively.

### Extraction of Bioactive Compounds and HPLC Analysis

The RAS samples were dried in the shade, pulverized into powder, and sieved through a 0.25 mm filter. The powder (0.5 g) was placed in a 150 ml vial containing 20 ml of 70% MeOH, sealed, weighed, and then sonicated at 220 W, 80 Hz for 40 min. Next, they were shaken with 70% MeOH, extracted, and verbed on a 0.22 μm microporous membrane. The filtered solution was stored at 4°C and quantified by HPLC using Agilent 1260 (Agilent, United States) and Merk RP-C18 (250 mm × 4.6 mm, 5 μm) devices. A mobile phase consisting of 0.1% glacial acetic acid (A) and acetonitrile (B) was used for separation with the following gradient: 0∼20 min: 19% B; > 20∼60 min: 19∼95% B; > 60∼75 min: 95∼100% B. The mobile phase flow rate was 1 mL/min, and the column temperature was 30°C. The detection wavelength was 280 nm.

### RNA Isolation, cDNA Library Construction, and Sequencing

Briefly, total RNA was digested using DNase. Then poly A-containing mRNA was enriched using oligo (dT)-attached magnetic beads, and then randomly fragmented into small segments. The first and second strands of cDNA were synthesized using the fragments as templates, and then end repair was done. The double-stranded cDNAs were purified with a QiaQuick PCR extraction kit (Qiagen) and eluted with EB buffer for end repair and poly (A) addition. Finally, sequencing adapters were ligated to the 5′ and 3′ ends of the fragments. The fragments were purified via agarose gel electrophoresis and amplified via PCR to create a cDNA library. The cDNA library was sequenced on an Illumina sequencing platform (Illumina HiSeq™2500) by Shanghai OE Biotech. Co., Ltd., Shanghai, China (Bolger et al., 2014).

### Bioinformatic Analyses

Raw data in fastq format were first filtered by removing reads containing adapters and/or ploy-N and other low-quality reads. The remaining clean data were assembled using the Trinity program (Grabherr et al., 2011). The functions of unigenes were annotated by aligning them with the NCBI non-redundant (NR), SwissProt, and Clusters of Orthologous Groups for Eukaryotic Complete Genomes (KOG) databases using Blastx (Altschul et al., 1990) with a threshold *E*-value of 10^–5^. Differential expression analyses were performed using the DESeq R package. A *P*-value < 0.01 with Foldchange ≥ 2.0-fold was set as the threshold for significant differential expression. KEGG enrichment analysis of differentially expressed genes (DEGs) was performed using R based on a hypergeometric distribution.

### qRT-PCR

Identical RNA samples as those used in the RNA-seq experiments were used for qRT-PCR. The relative expression patterns of six genes differentially expressed in transcriptome data were evaluated. Quantification was performed via a two-step reaction process: reverse transcription (RT) and PCR. Each RT reaction consisted of 0.5 μg RNA, 2 μL 5 × TransScript All-in-one SuperMix for qPCR and 0.5 μL gDNA Remover, in a total volume of 10 μL. Reactions were performed on a GeneAmp^®^ PCR System 9700 (Applied Biosystems, United States) for 15 min at 42°C, 5 s at 85°C. Then the 10 μL RT reaction mix was diluted × 10 in nuclease-free water and held at –20°C. The expression levels of mRNAs were normalized to the reference gene and were calculated using the 2-ΔΔCt method.

### Data Analysis

All data were analyzed using SPSS Statistics software (IBM, New York, United States). Differences in physiological response effects and bioactive compound levels between the two cultivars at different UV-B doses were analyzed via analysis of variance (ANOVA). The least significant difference (LSD) was calculated for significant data at *P* < 0.05. The reported values represent arithmetic averages of three replicates, and the data are expressed as mean ± standard deviation (SD).

## Results

### Biomass

**FIGURE 2:**
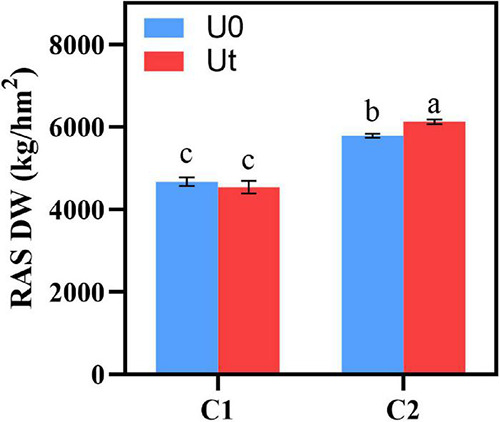
Biomass of two *Angelica sinensis* cultivars in response to enhanced UV-B radiation. Different letters indicated the significant difference (*P* < 0.05). Error bars were represented standard deviation (SD).

To evaluate the effect of enhanced UV-B radiation, the dry weight (DW) was measured in RAS after drying. DW of C2 was higher than that of C1 regardless of whether UV-B radiation enhancement was carried out, and DW of enhanced UV-B radiation was significantly higher than that of natural light (*P* < 0.05) (Figure 2).

### Photosynthesis Response

**FIGURE 3:**
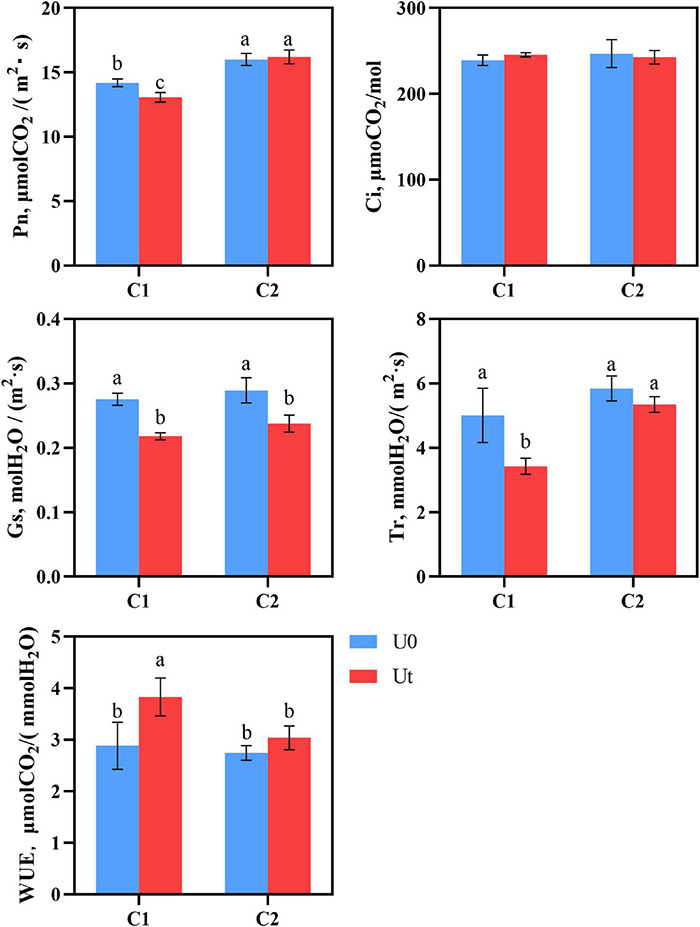
Photosynthesis of two *Angelica sinensis* cultivars in response to enhanced UV-B radiation. Different letters indicated the significant difference (*P* < 0.05). Error bars were represented standard deviation (SD).

As photosynthesis is one of the first processes affected by enhanced UV-B radiation, it is crucial to evaluate its response. There were significant differences in the photosynthesis parameters, including Pn, Ci, Gs, Tr, and WUE between C1 and C2 under each treatment. In C1, enhanced UV-B radiation significantly decreased Pn, Gs, and Tr by 8.3, 26.3, 46.1, and 33.0%, respectively, compared to natural light. In C2, Gs was significantly decreased by 17.2%. Pn was 1.24 times higher in C2 than in C1 under radiation. It can be speculated that C2 maintains its photosynthesis by closing stomata and reducing the loss of water dispersion (Figure 3).

### Photosynthetic Pigments

**FIGURE 4:**
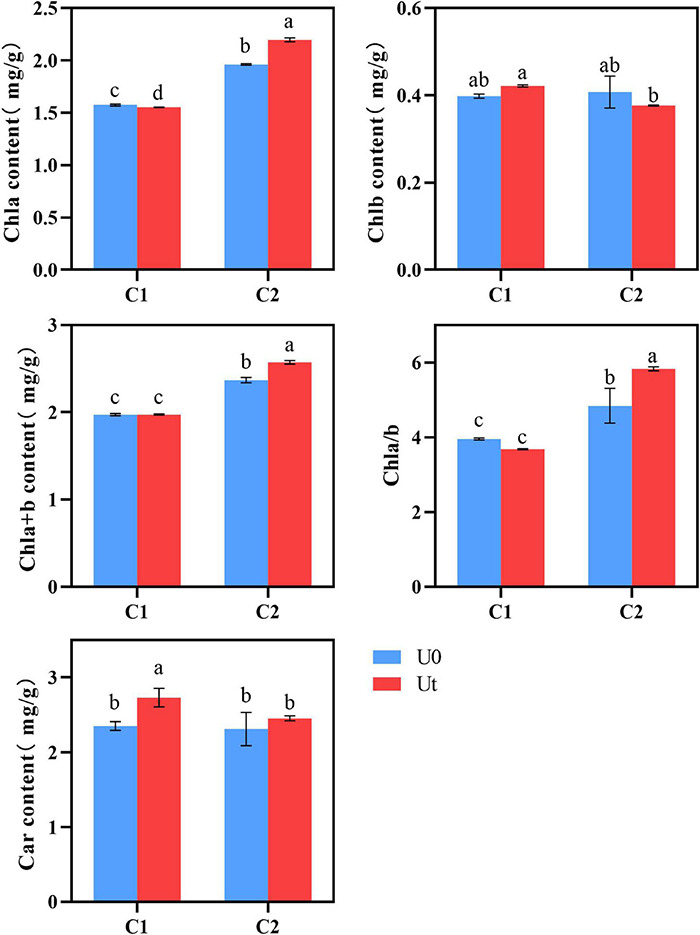
Photosynthetic pigment of two *Angelica sinensis* cultivars in response to enhanced UV-B radiation. Different letters indicated the significant difference (*P* < 0.05). Error bars were represented standard deviation (SD).

Chl content in plant leaves is an important physiological index to measure plant stress resistance (Ben-Asher et al., 2006). No differences were found in the photosynthetic pigments (Chl b, Chla + b, and Chl a/b) of C1, whereas Car was 1.11 times higher under radiation. In C2, radiation significantly affected all pigments except Chl b and Car, levels of Chl a, Chla + b, and Chla/b increased by 22.6, 7.1, and 20.5%, respectively, and Chlb decreased by 7.5%. It can be inferred that C1 can adapt to enhanced UV-B radiation by increasing levels of Car, a light absorption auxiliary pigment, clearing excess ROS in tissues, and protecting Chl. By contrast, the higher Chl a/b of C2 indicates that the thylakoid membrane structure of C2 was stable under enhanced UV-B radiation, it had better adaptability under radiation, and the accumulation of Chl content was somewhat promoted (Figure 4).

### Oxidative Damage and the Antioxidant Defense System

**FIGURE 5:**
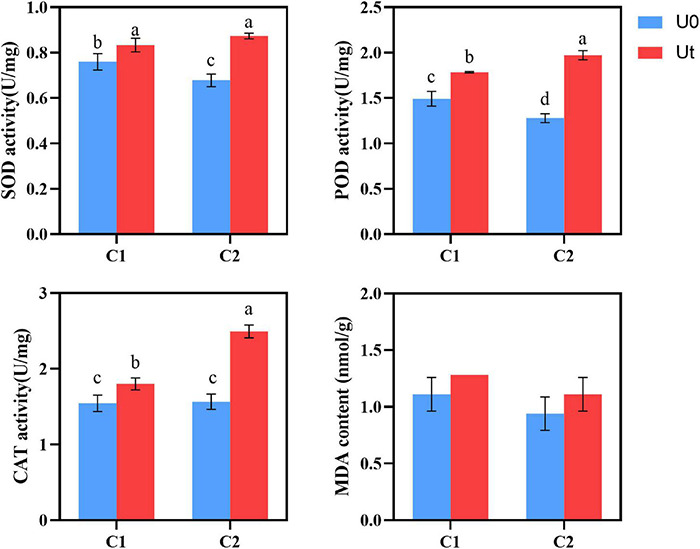
SOD, POD, and CAT activities and MDA content of two *Angelica sinensis* cultivars in response to enhanced UV-B radiation. Different letters indicated the significant difference (*P* < 0.05). Error bars were represented standard deviation (SD).

Compared to natural light, enhanced UV-B radiation increased the activities of antioxidant enzymes, such as SOD, POD, and CAT in leaves of *A. sinensis*. In detail, the increases were 9.7 and 28.8% for SOD, 20.1 and 53.9% for POD, and 16.9 and 59.6% for CAT in C1 and C2, respectively. To evaluate the effects of UV-B stress on membrane lipid peroxidation in leaves, MDA levels were measured. No significant differences were noted between the treatments. Plant tissues and cells protect against injuries derived from UV-B oxidative stress through the upregulation of multiple antioxidant enzymes. It shows that under enhanced UV-B radiation, increasing antioxidant enzyme activity (SOD, POD, and CAT) is the common adaptation method of C1 and C2, and the three protective enzymes can synergistically scavenge stress-induced ROS (Figure 5).

### Bioactive Compounds

**FIGURE 6:**
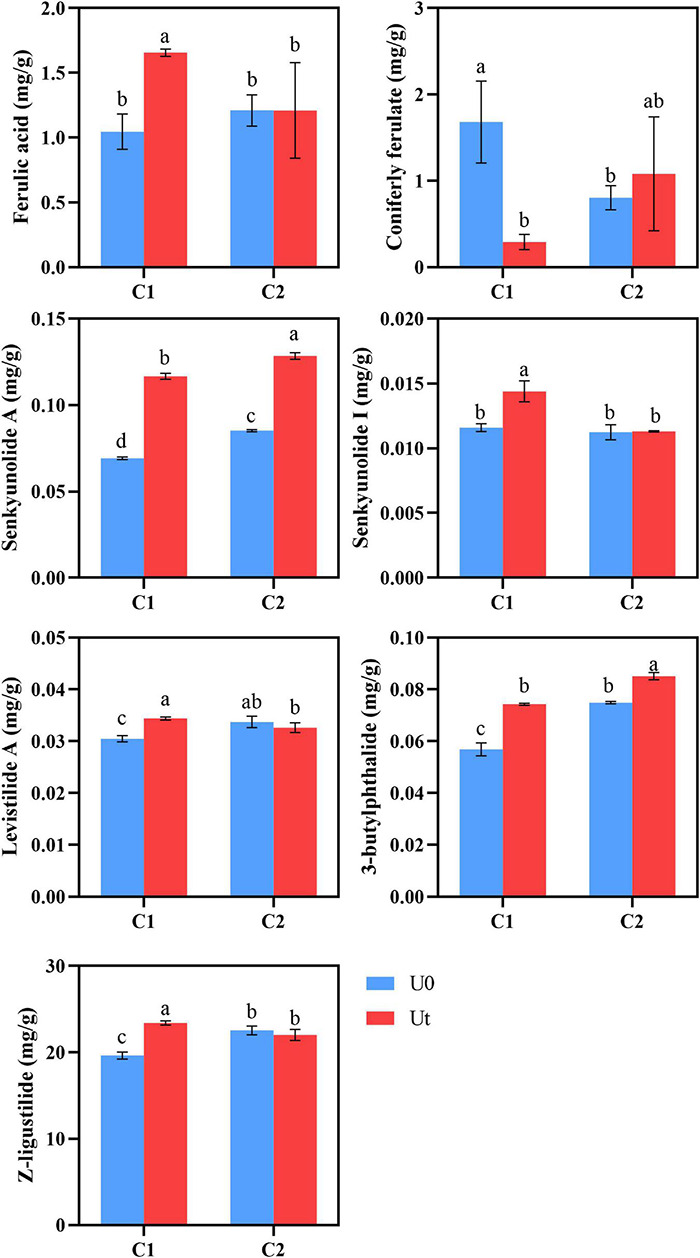
Levels of bioactive compounds of two RAS cultivars in response to enhanced UV-B radiation. Different letters indicated the significant difference (*P* < 0.05). Error bars were represented standard deviation (SD).

Radiation positively impacted the accumulation of senkyunolide A and 3-butylphthalide in both cultivars compared to natural light. It increased the content of Z-ligustilide by 19.27% and levistilide A by 9.7% in C1, and decreased the content of Z-ligustilide by 2.3% and levistilide A by 2.9% in C2. Senkyunolide I of C1 was significantly increased by 16.7%, but there were no significant differences in C2. Coniferyl ferulate can be regarded as the “reserve pool” of ferulic acid in RAS. Coniferyl ferulate can be easily decomposed into ferulic acid. Therefore, ferulic acid in RAS should be judged in combination with coniferyl ferulate. Generally speaking, C2 will not have a negative impact on the accumulation of ferulic acid content under enhanced UV-B radiation, on the contrary, C1 may be reduced. UV-B had a positive effect on the accumulation of C1 lactones (Figure 6).

### Identification and Validation of Differentially Expressed Genes

**FIGURE 7:**
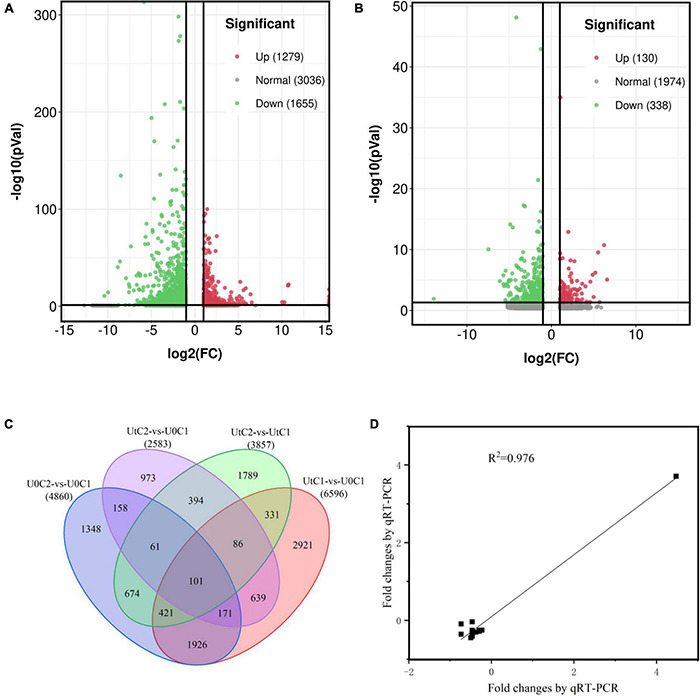
Annotation and differentially expressed genes (DEGs) of the *Angelica sinensis* transcriptome. **(A,B)** Volcanic plots of the upregulated and downregulated genes of *Angelica sinensis* in two cultivars under enhanced UV-B radiation and natural light. **(C)** Unigene Venn diagrams for each group. **(D)** Correlation analysis between qRT-PCR and RNA-seq results of six genes.

A total of 6596 DEGs (2851 upregulated and 3745 downregulated) were identified in C1 between treatments (UtC1 vs. U0C1). Only 2583 DEGs (822 upregulated and 1761 downregulated) were found in C2 (UtC2 vs. U0C2). The number of DEGs caused by radiation was 3603 and 2600 in C1 and C2, respectively. The number of DEGs shared by the two cultivars was 1257 (Figures 7A–C).

To verify the sequencing data, we selected six unigenes for qRT-PCR verification. Of these, several unigenes encoding *psaH*, *SAUR*, *FAH*, *Lhca4*, *WIN1*, and *C2H2* were experimentally validated by quantitative RT-PCR, and there was good concordance (*R*^2^ = 0.976) between RNA-seq data and qRT-PCR analysis (Figure 7D), indicating that the gene expression levels were reliable. The primers are shown in Supplementary Table 1.

### Kyoto Encyclopedia of Genes and Genomes Pathway Analysis of Differentially Expressed Genes

**FIGURE 8:**
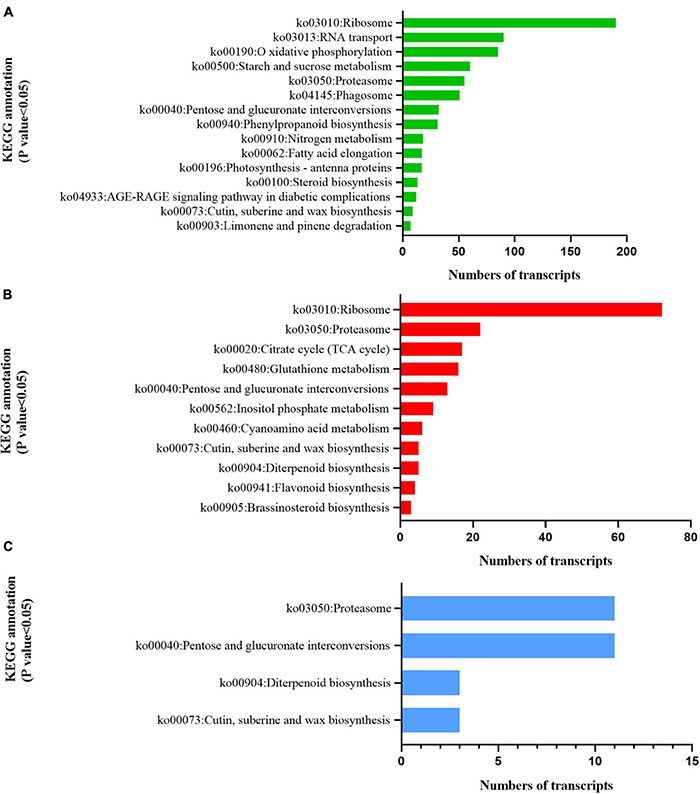
Kyoto Encyclopedia of Genes and Genomes (KEGG) enrichment analysis of pathways involved in the response to enhanced UV-B radiation. **(A)** Mingui 1, **(B)** Mingui 2, **(C)** common pathways of two cultivars.

KEGG analysis illustrated that the two cultivars responded differently to radiation. DEGs in C1 were significantly enriched in photosynthetic antenna protein (ko00196), indicating that radiation had a significant effect on light-harvesting ability (Jobe et al., 2019; Figure 8A). C2 was significantly enriched in the TCA cycle (ko00020), glutathione metabolism (ko00480), inositol phosphate metabolism (ko00562), cyanoamino acid metabolism (ko00460), flavonoid biosynthesis (ko00941), and brassinosteroid biosynthesis (ko00905) pathways (Figure 8B). This indicates that radiation widely affected the synthesis and accumulation of primary and secondary metabolites. Both C1 and C2 were enriched in the cutin, suberine, and wax biosynthesis pathways (ko00073), indicating that UV-B affects the production of wax and lignin on the leaf surface (Figure 8C).

### Differentially Expressed Genes Related to the Photosynthetic System

**FIGURE 9:**
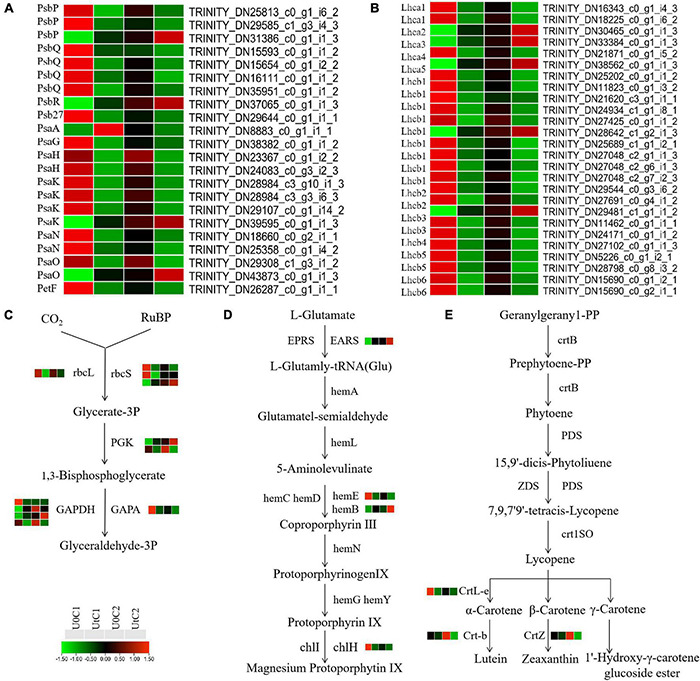
Heat maps of differentially expressed genes (DEGs) in Mingui 1 and Mingui 2 leaves. **(A)** Photosynthesis, **(B)** photosynthetic-antenna protein, **(C)** carbon fixation, **(D)** chlorophyll metabolism, **(E)** carotenoid biosynthesis. Heat maps were drawn using log2-transformed FPKM values.

KEGG enrichment analysis revealed that photosynthetic systems in C1 and C2 responded differently to enhanced UV-B radiation. A total of 48 DEGs between natural light and irradiation conditions were annotated to three pathways: 22 for photosynthesis (ko00195), 17 for photosynthesis-antenna proteins (ko00196), and 9 for carbon fixation (ko00710). These DEGs were concentrated in C1. In total, 22 genes (17 downregulated, 5 upregulated) participated in the photosynthetic reaction center including the PSI (*Psa A*, *Psa G*, *Psa H*, *Psa K*, *Psa N*, and *Psa O*) and PSII complexes (*Psb P*, *Psb Q*, *Psb R*, and *Psb 27*) and photosynthetic electron transport (*PetF*) in C1. One gene encoding *Psa H* and one encoding *Psa O* were downregulated in the photosynthetic reaction center in C2 (Figure 9A). In C1, *Lhca3* was significantly up-regulated, and one lhca1 coding gene and one *Lhcb2* coding gene were up-regulated. In C2, *Lhca4*, *Lhcb2*, and *Lhcb6* were significantly downregulated, although overall the number of downregulated genes was much lower than in C1 (Figure 9B). Among the photosynthetic-related pathways in KEGG, only the photosynthetic antenna protein pathway was significantly downregulated in C1 (Figure 9C). However, there is no significant enrichment of photosynthetic system related pathways in C2. In C1, under UV-B enhanced radiation, most of the nine coding DEGs of the key enzymes *rbcL*, *rbcs*, *PGK*, *GAPDH*, and *GAPA* in the photosynthetic biological carbon fixation pathway were down-regulated. In C2, the expression of related genes in the photosynthetic system is stable (Figure 9D). However, the photosynthetic system of C1 is more sensitive to enhanced UV-B radiation, most DEGs are downregulated, and photosynthesis may be adversely affected.

The adaptability of plants to UV-B stress is inextricably related to chlorophyll metabolism (ko00860) and carotenoid biosynthesis (ko00960). One gene encoding *EARS* was significantly upregulated, and one gene encoding *hemE* and one encoding *chlH* were downregulated after radiation exposure in C1. Only one gene encoding *hemB* was upregulated in C2. In addition, one gene of *CrtL-e* was downregulated after radiation in C1. One gene of *Crt-b* and one gene of *CrtZ* were downregulated after radiation in C2 (Figure 9E). In conclusion, enhanced UV-B radiation was more beneficial for the accumulation of photosynthetic pigments in C2 than in C1.

### Differentially Expressed Genes Involved in Defense Responses

#### Antioxidant System

**FIGURE 10:**
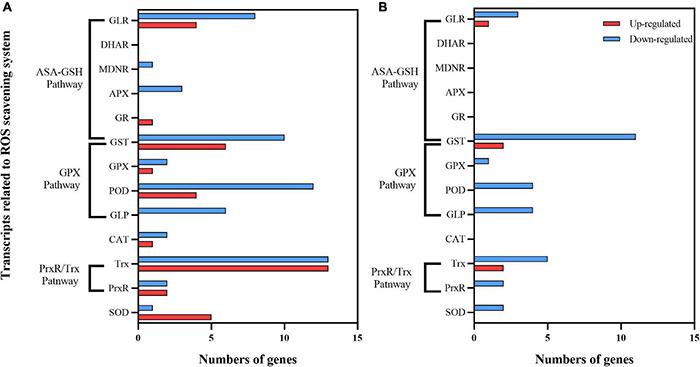
ROS scavenging system of **(A)** Mingui 1 and **(B)** Mingui 2 under enhanced UV-B radiation.

UV-B stress causes the accumulation of ROS in plants (Hideg et al., 2013). ROS scavenging enzymes such as SOD, CAT, POD, and ascorbate peroxidase (APX), as well as non-enzymatic antioxidants (glutathione, carotenoids, etc.) After 30 days of exposure to enhanced UV-B radiation, there were 97 DEGs in C1 (30 upregulated, 60 downregulated). Among up-regulated genes, 11 ASA-GSH cycle-related genes (6 GSTs, 1 GR, and 4 GLRS), 11 GPX-related pathway genes (4 PODS, 1 GPX, and 6 GLRS), 15 PrxR/Trx pathway-related coding genes (2 PrxR, 13 Trx), and 5 SOD encoding genes were identified (Figure 10A). There were only 5 upregulated and 32 downregulated DEGs in C2 (Figure 10B). The upregulated DEGs in both cultivars were focused on the AsA-GSH cycle and PrxR/Trx pathways.

#### Transcription Factors

**FIGURE 11:**
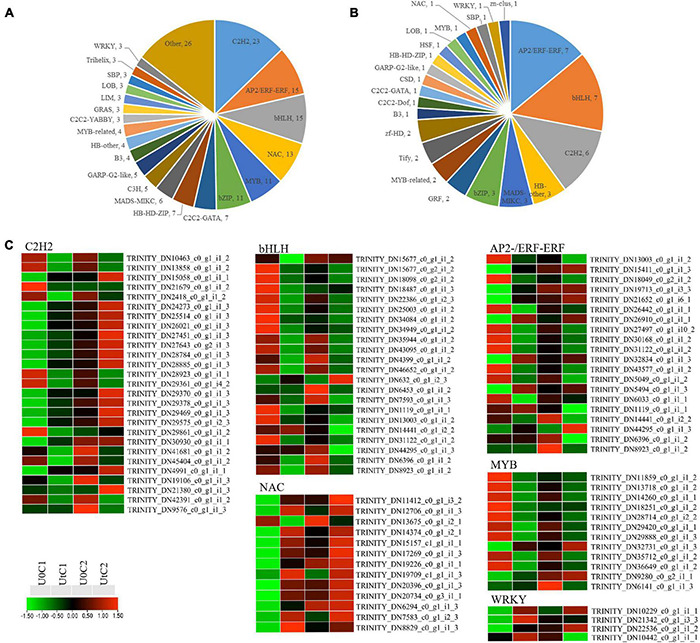
Differentially expressed genes (DEGs) related to transcription factor (TF) families in *Angelica sinensis*. **(A)** Mingui 1, **(B)** Mingui 2, **(C)** Heatmap.

A total of 177 and 50 TF-encoding genes were differentially expressed in irradiated C1 and C2 (and classified into 39 and 23 families), respectively, compared to natural light (Figures 11A,B). The largest number of such genes identified belonged to the TF families *C2H2* (27), *bHLH* (22), and *AP2/ERF-ERF* (21), making up more than 36.08% of the total number of TFs. Among *C2H2* and *AP2/ERF-ERF* TFs, about half were upregulated and half downregulated after radiation exposure in C1. However, these were mostly downregulated in C2. In C1, all *WRKY* (*WRK72*, *WRK50*, and *WRK22*) and one heat shock TF (*HSF*) were significantly upregulated after irradiation. In the *NAC* family, except for *NAC* domain-containing protein 41, the remaining 12 coding genes were also upregulated, but *bHLH* families were significantly downregulated after irradiation. MYB TFs are positive regulators of flavonoid biosynthesis, and two genes encoding *MYB78* were upregulated; the remaining encoding genes were downregulated after radiation exposure in C1. In C2, one coding gene each was downregulated after radiation for *WRKY*, *MYB*, and *HSF*, namely, *WRK15*, *MYB73*, and *cytochrome P450 711A1*, respectively. In addition, one *NAC* family gene was upregulated (Figure 11C).

#### Phytohormone Signaling

**FIGURE 12:**
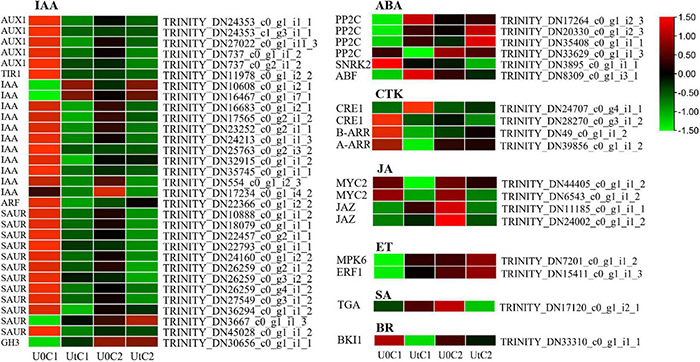
Heatmap of differentially expressed genes (DEGs) related to plant hormone signal transduction of Mingui 1 and Mingui 2. Heat maps were drawn using log2-transformed FPKM values.

Phytohormone signaling plays a key role in plant development, and hormones such as IAA, ethylene (ET), brassinosteroid (Br), ABA, jasmonic acid (JA), salicylic acid (SA), and cytokinins (CTK) are deeply involved in the regulation of plant morphology and metabolic responses (Figure 12). C1 had 44 DEGs (11 upregulated, 33 downregulated) in pathways related to phytohormones, and 30, 4, 5, 2, 1, and 2 DEGs in the IAA, CYT, ABA, ET, Br, and JA pathways, respectively. C2 had 7, 2, 2, and 1 DEG in the IAA, ABA, JA, and SA pathways, respectively, and most were downregulated after radiation treatment. In both cultivars, most DEGs were in the IAA signal transduction pathway. In this pathway, there were four times more downregulated IAA genes than upregulated ones. The *GH3*, *PP2C*, and *ABF* genes were significantly upregulated in C1, while one *PP2C* gene was upregulated and one was downregulated in C2. These results showed that in C1, *MPK6* and *ERF1/2* were significantly upregulated in ET signaling pathway, *A-ARR* and *B-ARR* were significantly downregulated in ZT signaling pathway, and *BKI1* and *MYC2* were downregulated in BR and JA signaling pathway. In C2, in JA and SA pathways, the expression of *JAZ* and *TGA* were significantly downregulated.

### Differentially Expressed Genes Related to Secondary Metabolism

#### Ferulic Acid Biosynthesis

**FIGURE 13:**
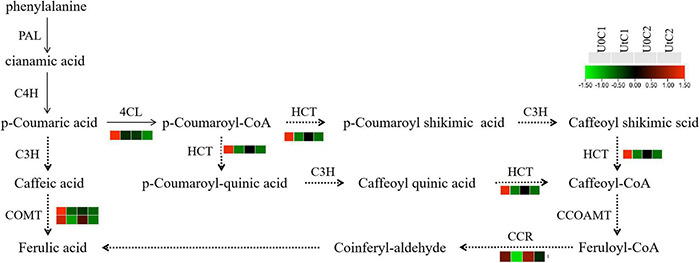
Heatmap of differentially expressed genes (DEGs) related to ferulic acid synthesis in Mingui 1and Mingui 2. Heat maps were drawn using log2-transformed FPKM values.

Ferulic acid has antioxidant, antibacterial, and anti-inflammatory effects, among many other beneficial features, and is internationally recognized as an anti-cancer substance. It is easily absorbed by the human body and can be metabolized and excreted from urine. It has low toxicity and is relatively safe to use. Its medicinal value has attracted increasing attention (Sitarek et al., 2017). To further explore the metabolic mechanism of ferulic acid under enhanced UV-B radiation, its pathway and the expression level of related genes were visualized. KEGG data showed that the phenylpropanoid biosynthesis pathway was significantly enriched in C1, with a total of 31 DEGs (5 upregulated, 26 downregulated); there were only 9 DEGs in this pathway in C2. The key enzymes were *4CL*, *HCT*, *C3H*, *PAL*, *C4H*, *CCR*, *COMT*, and *CCOAMT*; the *4CL*, *CCR*, and *HCT* genes were all downregulated in C1. Only one gene encoding *COMT* was downregulated in C2 after radiation treatment in genes related to these key enzymes (Figure 13).

#### Lactone Biosynthesis

**FIGURE 14:**
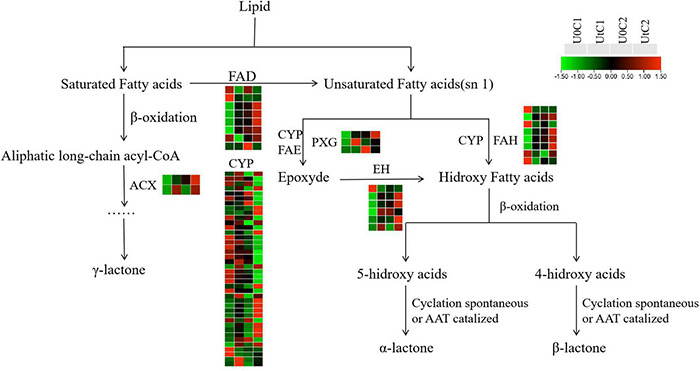
Heatmap of differentially expressed genes (DEGs) related to lactone biosynthesis in Mingui 1 and Mingui 2. Heat maps were drawn using log2-transformed FPKM values.

New evidence suggests that Z-ligustilide has a wide range of pharmacological properties, including anticancer, anti-inflammatory, antioxidant, and neuroprotective activities (Donkor et al., 2016; Xie et al., 2020). Figure 14 shows the changes in key enzymes (*CYP*, *FAD*, *EH*, *ACX*, *PXG*) in lactone synthesis in C1 and C2 under irradiation, compared to natural light. The number of DEGs was much lower in C2 than in C1, and the number of upregulated DEGs was much higher in C1 than in C2. In this synthetic pathway, most DEGs encoded *CYP* in C1 (20 upregulated, 27 downregulated) and C2 (3 upregulated, 14 downregulated). Both *ACX* and *PXG* were significantly upregulated in C1, while only one coding gene was downregulated in *EH*; the rest were also significantly overexpressed. In C1, the upregulated number of DEGs encoding *FAD* and *FAH* was close to the downregulated number, while in C2, all DEGs encoding *FAD* were down-regulated. We conclude that the accumulation of lactones has a positive effect on C1 and may be neutral or has a slight adverse effect on C2.

#### Flavonoid Biosynthesis

**FIGURE 15:**
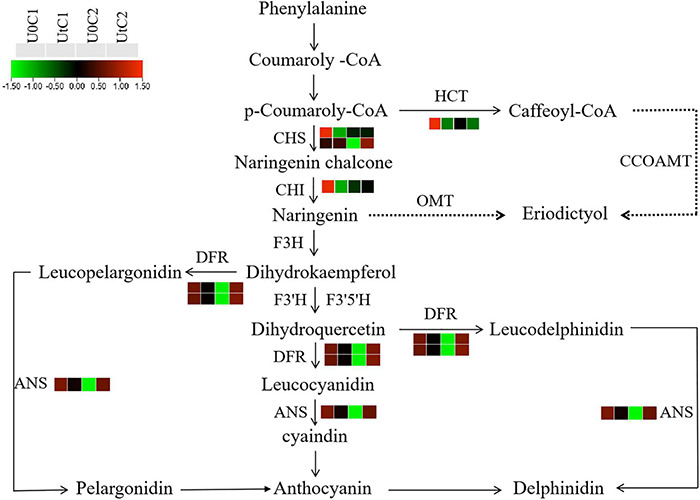
Heatmap of differentially expressed genes (DEGs) related to flavonoid biosynthesis in Mingui 1 and Mingui 2. Heat maps were drawn using log2-transformed FPKM values.

Flavonoids have analgesic, hypotensive, anticoagulant, and cerebrovascular protective effects (Lovegrove et al., 2017). After irradiation, C2 was significantly enriched in flavonoid biosynthesis, with dihydroflavonol 4-reductase (*DFR*), chalcone synthase (*CHS*), and anthocyanin synthase (*ANS*) being significantly upregulated. In C1, three DEGs were significantly downregulated in this pathway, including *CHS*, chalcone isomerase (*CHI*), and shikimate o-hydroxycinnamoyl transferase (*HCT*). Therefore, after irradiation, more flavonoids were accumulated in the leaves of C2 (Figure 15).

## Discussion

### Photosynthesis Response

Plants can adapt to different environmental stresses by changing gene expression. In our study, PSI and PSII of C1 were more susceptible to enhanced UV-B radiation. The peripheral subunits (*PsbP*, *PsbQ*, and *PsbR*) of the oxygen-evolving complex (OEC) responsible for the stabilization of PSII were differentially expressed after radiation treatment, whereas most DEGs were downregulated. *PsbR* plays an important role in polymerization stability, photosynthetic protection, and water oxidation. It may be that its coding gene was significantly upregulated and maintained the efficiency of PS II in C1 (De Las Rivas et al., 2007). *Psb27* protein is one of the most important assembly and repair factors. It plays an important role in maintaining efficient assembly and repair of PSII under stresses such as low temperature, high light, and a rapid change in light intensity (Roose and Pakrasi, 2008). One coding gene of *Psb27* was significantly downregulated in C1. *PsaA* is the most important protein in PS I, and membrane mosaic proteins such as *PsaG*, *PsaH*, *PsaK, PsaN*, and *PsaO* surround its core. In C1, the upregulation of *PsaA* plays an important role in maintaining PSI function (Jagannathan and Golbeck, 2009). In our study, the photosynthetic system of C2 was stable under enhanced UV-B radiation, and only one coding gene of *PsaH* and *PsaO* was downregulated in PS I. This indicates that enhanced UV-B radiation has a negative effect on the transfer of light energy from PS II to the center of PS I. The photosystem is composed of a photosynthetic reaction center and a peripheral antenna. Antenna proteins are the most important part of the light-harvesting complex (LHC) in photosynthesis. Under irradiation, the photosynthesis antenna protein pathway of C1 was significantly enriched, and the expression of several genes encoding *Lhcb* and *Lhca* were downregulated. This indicates that UV-B stress may affect the LHC of *A. sinensis*, consistent with previous experimental studies (Khudyakova et al., 2019). There was no significant enrichment in this pathway for C2.

### Antioxidant System

To protect plants from ROS, plants have evolved a self-protection system that can eliminate ROS by combining antioxidant enzymes and small-molecule antioxidants (Fan et al., 2021). The ASA-GSH cycle and PrxP/TRX pathway play an important role in the resistance of the two varieties to UV-B stress. The number of DEGs was lower in C2 than in C1. These results indicate that the activities of antioxidant enzymes in leaves increased under enhanced UV-B radiation, which maintained the ability of plants to scavenge ROS and prevented damage to leaves, thus preserving photosynthetic efficiency. These results support the results of gene expression levels.

### Transcription Factors

The expression of plant TFs is typically quickly altered in response to environmental stimuli, leading to the establishment of a pleiotropic phenotype to enhance stress tolerance (Joshi et al., 2016). Enhanced UV-B radiation affects phenolic metabolic pathways, as well as the levels of various phenols, by regulating the expression of various genes in the bHLH-MYB-WD40 complex (Zhou et al., 2015). In our study, almost all *bHLH* and *MYB* coding genes were downregulated, which shows that phenols had a poor response to enhanced UV-B radiation in C1. Among these TFs, *WRKY* is one of the most important in higher plants, as it participates in pathogen defense responses (Shi et al., 2020). *Arabidopsis AtWRKY22* and *AtWRKY29* proteins are essential components of MAPK-mediated plant defense responses against pathogens (Asai et al., 2002). Knockout of *WRKY22* enhances susceptibility to *Magnaporthe oryzae*, and overexpression of *WRKY22* enhances resistant phenotypes in rice (Abbruscato et al., 2012). *Arabidopsis AtWRKY50* might function as a positive regulator of the SA-mediated signaling pathway and a negative regulator of the JA acid-mediated signaling pathway, thereby enhancing resistance to gray mold disease (Gao et al., 2011). *WRKY72* and its *Arabidopsis* homolog *AtWRKY72* are similarly involved in the pathogen defense process of *Meloidogyne* and Oomycota, and this process is most likely independent of the SA pathway (Bhattarai et al., 2010). In our study, *WRKY22*, *WRKY50*, and *WRKY72* were highly expressed in C1. *NAC* TFs are ubiquitous in plants, and their expression significantly changes under abiotic stresses (Yang et al., 2015). In our dataset, the expression of the *NAC* TFs genes was upregulated under enhanced UV-B radiation, compared to natural light. The involvement of *NAC* TFs in UV-B tolerance has been demonstrated in various crops such as wheat. Some less common TFs such as C2H2-zinc finger proteins are also essential in plant stress responses, although their transcriptional regulatory mechanisms remain largely unclear. In addition, one *HSF* with high expression was detected in C1 under enhanced UV-B radiation, consistent with previous studies that *HSF* plays a crucial role in protecting against oxidative stress. This suggests that the regulation of development and metabolism by TFs is more prominent in C1. The details of the interactions among these genes and how they function require further research.

### Phytohormone Signaling

Plant hormones play a key role in abiotic tolerance by mediating a wide range of responses. In this study, under enhanced UV-B radiation, the number of DEGs involved in the hormone signaling pathway was far less in C2 than in C1. Auxin and gibberellin are growth-promoting molecules. In response to auxin, many genes were downregulated, but the expression of DEGs encoding IAA was higher in C2 than C1. Biomass of C1 did not change significantly, it may be that the *GH3* gene was significantly upregulated and the *GH3* protein participated in the binding of free IAA and amino acids, thus controlling auxin homeostasis, while *GH3* gene was not found in C2, so the activity of IAA under enhanced UV-B radiation was higher than that of under natural light, resulting in a significant increase in its biomass (Feng et al., 2019). ABA, SA, and JA were mainly involved in the stress response and adaptation. *PP2C* and *ABF* were significantly upregulated in C1, and may thus constitute its adaptive response to UV-B stress. Under enhanced UV-B radiation, there were few genes involved in the response of each hormone in C2, indicating that this cultivar was stable under irradiation.

### Bioactive Compounds

The absorbance of flavonoids is 280∼340 nm, which is equivalent to that of sunscreen (Tienaho et al., 2021). It can act as a UV filter in plants to prevent rays from penetrating tissues, to protect the photosynthetic system from damage (McLay et al., 2020). In our study, *DRF* and *CHS* were the key enzymes in flavonoid biosynthesis, which were significantly upregulated in C2. The upregulation of key enzymes in the flavonoid biosynthesis pathway has a positive effect on the accumulation of flavonoids. Given the function of flavonoids in scavenging and absorbing UV-B, it is speculated that C2 has strong resistance to enhanced UV-B radiation. The secondary metabolism gene of ferulic acid in RAS may promote blood circulation and prevent blood stasis (Yuan et al., 2019). In our study, there was no significant difference in the gene expression of enzymes related to ferulic acid synthesis in C2, while *4CL*, *CCR*, and *COMT* were downregulated in C1. This indicates that C1 is less favorable to the accumulation of ferulic acid under enhanced UV-B radiation. In this pathway, the *COMT* gene can also regulate the synthesis of lignin, and an increase in the content of lignin is conducive to the response to UV-B stress. Despite the importance of lactones for RAS quality, there is a lack of information on the enzymes and genes associated with their biosynthesis. Nevertheless, it seems clear that lactone biosynthesis starts from fatty acids with the introduction of an O atom to form hydroxy fatty acids. In our study, most DEGs were highly expressed in C1. *ACX* is widely involved in embryo development, seed germination, seedling establishment, and the biosynthesis of JA in response to stresses (Baker et al., 2006). Plant *cytochrome P450* is a monooxygenase, in the process of plant growth and development, P450 participates in secondary metabolic reactions such as the synthesis and degradation of alkaloids, terpenoids, fatty acids, plant hormones, signal molecules, and flavonoids (Cheng et al., 2020). In addition, it also plays an important role in the response mechanism to biological and abiotic stresses. *PXG* can protect plants from peroxyhydroxy fatty acids produced by lipoxygenase (*LOX*) enzymatic reactions and stress, and can also be transformed into derivatives to prevent fungal infection. The vast majority of genes encoding *PXG*, *CYP*, and *ACX* were upregulated in C1 in the present study. Under enhanced UV-B stress, the accumulation of lactones may have a positive effect in C1, leading to strong disease resistance. It may be neutral or have a slight adverse effect in C2.

## Conclusion

Resistance to UV-B radiation is a complex phenomenon, often a fusion of multiple adaptive properties. The tolerance mechanisms include an increase in photosynthetic pigment levels, enhancement of antioxidant enzyme activity, accumulation of plant growth hormones, and an increase in phenolic and flavonoid levels.

In the present study, Pn, levels of photosynthetic pigments (Chl a, Chl b, and Car), antioxidant enzyme activities (SOD, POD, and CAT), and accumulation of bioactive substances (ferulic acid and flavonoid) were maintained or were enhanced in C2, and MDA accumulation was stable under enhanced UV-B radiation. It can be proved that enhanced UV-B radiation has a positive regulatory effect on C2 growth. C2 outperformed C1.

We identified the genes involved in the response to enhanced UV-B radiation. They are involved in photosynthesis and photosynthetic pigment biosynthesis, TFs and ROS scavenging systems, hormone biosynthesis and signal transduction, and biosynthetic pathways of ferulic acid, lactones and flavonoids. The results were consistent with the differences in physiological and biochemical indexes. C1 has poor resistance to enhanced UV-B stress, while C2 has a positive regulatory effect on it. Finally, a large number of *A. sinensis* transcriptome data generated here will be used as a source to find effective methods to alleviate UV-B enhancement, and also help to improve the lack of genetic information of non-model plant species.

## Data Availability Statement

The datasets presented in this study can be found in online repositories. The names of the repository/repositories and accession number(s) can be found below: https://www.ncbi.nlm.nih.gov/bioproject/PRJNA767269.

## Author Contributions

YW designed the project and performed the literature research. TP acquired the main data, performed the statistical analysis, and edited the manuscript. TY, FW, JL, and YZ participated in the research and analyzed the data. All authors read and approved the final manuscript.

## Conflict of Interest

The authors declare that the research was conducted in the absence of any commercial or financial relationships that could be construed as a potential conflict of interest.

## Publisher’s Note

All claims expressed in this article are solely those of the authors and do not necessarily represent those of their affiliated organizations, or those of the publisher, the editors and the reviewers. Any product that may be evaluated in this article, or claim that may be made by its manufacturer, is not guaranteed or endorsed by the publisher.

## Abbreviations

ABA: abscisic acid
ACX: acyl-CoA oxidase
BR: brassinosteroid
CHS: chalcone synthase
CTK: cytokinin
CYP: cytochrome P450
DEG: differentially expressed gene
DFR: dihydroflavonol 4-reductase
EH: epoxide hydrolase
ET: ethylene
F3H: flavanone-3-hydroxylase
FAD: fatty acid desaturase
FAE: fatty acid epoxydase
FAH: fatty acid hydroxylase
FPKM: fragments per kilobase million
GA: gibberellin
GSH: glutathione
GST: glutathione s-transferase
HCT: shikimate O-hydroxycinnamoyl transferase
IAA: indole acetic acid
JA: jasmonic acid
KEGG: Kyoto Encyclopedia of Genes and Genomes
LOX: lipoxygenase
MDA: malondialdehyde
NR: non-redundant protein sequences
PAL: phenylalanine ammonia-lyase
PXG: peroxygenase
RAS: Radix Angelica Sinensis
ROS: reactive oxygen species
SA: salicylic acid
SD: standard deviation
TF: transcription factor
UV-B: Ultraviolet-B.
