# Supplementary figures and images for: Physiological and Biochemical Responses, and Comparative Transcriptome Profiling of Two Angelica sinensis Cultivars Under Enhanced Ultraviolet-B Radiation
Source: Front Plant Sci. 2021 Dec 17;12:805407. doi: 10.3389/fpls.2021.805407 (PMC8718920; doi:10.3389/fpls.2021.805407)

## Slide 1
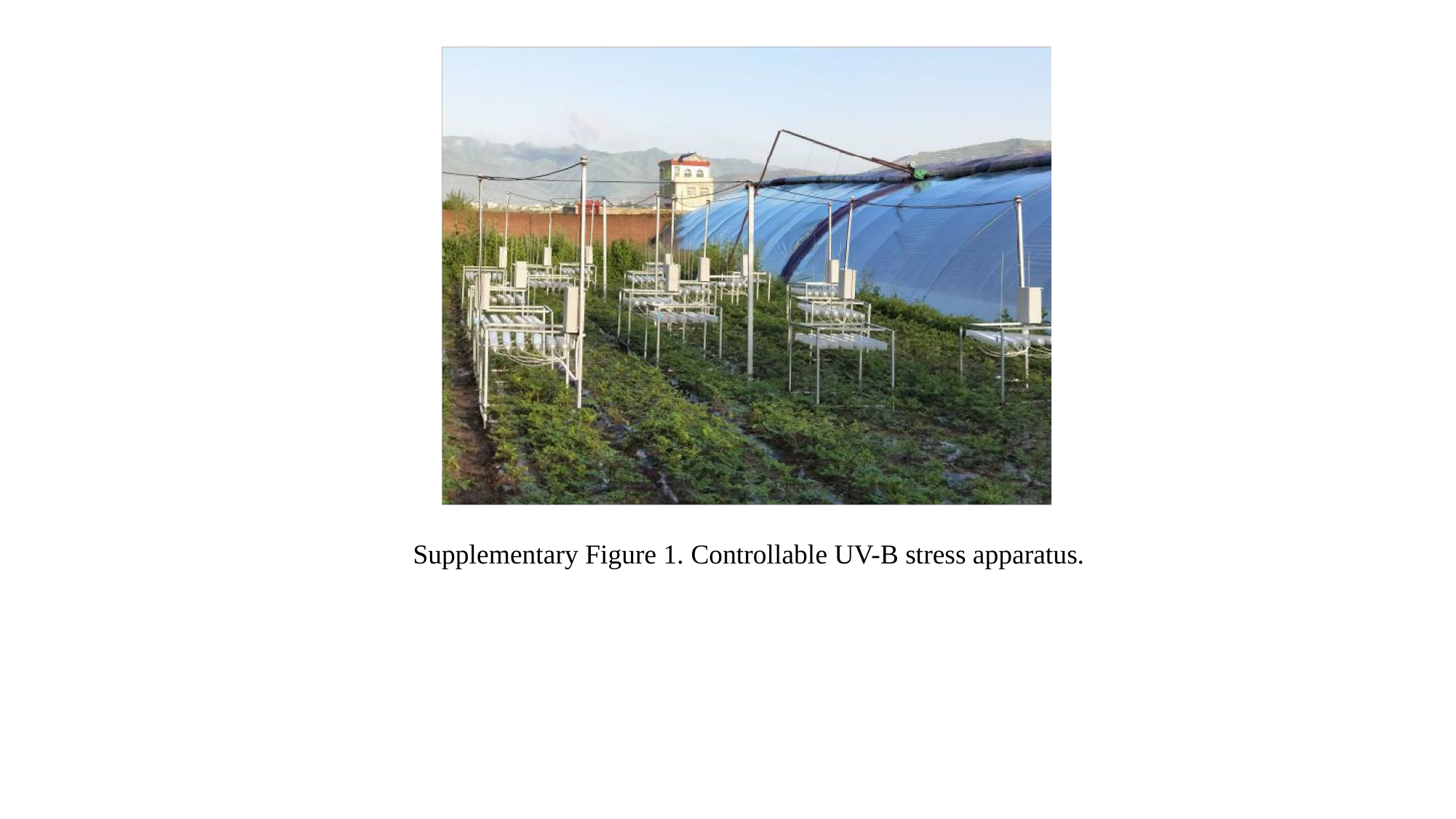

Supplementary Figure 1. Controllable UV-B stress apparatus.

Supplement: Supplementary file 2 [file Presentation_1.pptx]
